# Goat-associated Q fever: a new disease in Newfoundland.

**DOI:** 10.3201/eid0703.010308

**Published:** 2001

**Authors:** T F Hatchette, R C Hudson, W F Schlech, N A Campbell, J E Hatchette, S Ratnam, D Raoult, C Donovan, T J Marrie

**Affiliations:** Dalhousie University, Halifax, Nova Scotia, Canada. tom.marrie@ualberta.ca

## Abstract

In the spring of 1999 in rural Newfoundland, abortions in goats were associated with illness in goat workers. An epidemiologic investigation and a serologic survey were conducted in April 1999 to determine the number of infections, nature of illness, and risk factors for infection. Thirty-seven percent of the outbreak cohort had antibody titers to phase II Coxiella burnetii antigen >1:64, suggesting recent infection. The predominant clinical manifestation of Q fever was an acute febrile illness. Independent risk factors for infection included contact with goat placenta, smoking tobacco, and eating cheese made from pasteurized goat milk. This outbreak raises questions about management of such outbreaks, interprovincial sale and movement of domestic ungulates, and the need for discussion between public health practitioners and the dairy industry on control of this highly infectious organism.


### Goat-Associated Q Fever: A New Disease in Newfoundland

**Todd F. Hatchette,* Robert C. Hudson,† Walter F. Schlech,* Nancy A. Campbell,* Jill E. Hatchette,* Sam Ratnam,‡ Didier Raoult,§ Catherine Donovan,‡ and Thomas J. Marrie¶**

*Dalhousie University, Halifax, Nova Scotia, Canada; †Newfoundland Department of Forest Resources and Agrifoods, Clarenville, Newfoundland, Canada; ‡Memorial University of Newfoundland, St. John's, Newfoundland, Canada; §Unite des Rickettsies, Marseille, France; and ¶University of Alberta, Edmonton, Alberta, Canada

In the spring of 1999 in rural Newfoundland, abortions in goats were associated with illness in goat workers. An epidemiologic investigation and a serologic survey were conducted in April 1999 to determine the number of infections, nature of illness, and risk factors for infection. Thirty-seven percent of the outbreak cohort had antibody titers to phase II *Coxiella burnetii* antigen >1:64, suggesting recent infection. The predominant clinical manifestation of Q fever was an acute febrile illness. Independent risk factors for infection included contact with goat placenta, smoking tobacco, and eating cheese made from pasteurized goat milk. This outbreak raises questions about management of such outbreaks, interprovincial sale and movement of domestic ungulates, and the need for discussion between public health practitioners and the dairy industry on control of this highly infectious organism.

*Coxiella burnetii* is an obligate intracellular pathogen known to be the causative agent of Q fever, a zoonosis with a worldwide occurrence (1). The organism has been found in many wild and domestic animals (1-3). The most common reservoirs of infection in humans are domestic farm animals such as cattle, goats, and sheep (4-6). *C. burnetii* is shed in urine, feces, and milk from infected animals and has a particularly high concentration in products of conception (7). The organism is highly infectious: Only one organism is required to produce infection under experimental conditions (8,9). Inhalation of aerosolized microorganisms is thought to be the most important route of infection in humans. However, ingestion of raw milk products has also been implicated (6).

Although *C. burnetii* can cause abortion and stillbirth, most animals have a persistent, relatively asymptomatic subclinical infection (10). Infection in humans usually manifests as a self-limiting febrile illness, pneumonia, or hepatitis (11). Most patients have an uneventful recovery; however, chronic infections such as Q-fever endocarditis and chronic hepatitis are uncommon but well-documented sequelae (12).

The diagnosis of Q fever is usually established by demonstrating seroconversion to *Coxiella* antigens in conjunction with an appropriate clinical history (13). *C. burnetii* can have two distinct antigenic presentations or phases; animals and humans develop antibody responses to both phases. In humans, phase II gives rise to the predominant antibody response in acute infection, while response to phase I antigen is dominant during chronic infections (14).

In the spring of 1999, abortions were noted in goats on one farm belonging to a newly formed cooperative in rural Newfoundland. Aborted placenta had histologic evidence of *C. burnetii* infection. At the same time a number of farmers and their workers had a nonspecific febrile illness associated with severe headaches. Serologic testing revealed that these persons had recent infection with *C. burnetii*. No documented case of Q fever had previously been reported in Newfoundland. An epidemiologic investigation and serologic survey were started in April 1999 to determine the extent of the outbreak in animals and humans, the nature of the clinical illness, and risk factors for Q fever associated with this outbreak.

### Methods

### Identification of Cases

The cooperative consisted of eight goat farms within a 170-km$^2$ area of rural Newfoundland, with a population of approximately 8,000 people (Figure 1). In April 1999, farmers, workers, and contacts (family members of the farmers or workers and other persons who may have had contact with the farms) were interviewed by using a detailed questionnaire. Workers included persons who were involved directly with animal care as well as carpenters and other farm laborers. Serum samples were drawn to determine the presence of antibodies to *C. burnetii*. Family physicians in the area submitted serum samples from all patients in their practices who had been seen with symptoms compatible with Q fever.

The diagnosis of acute *C. burnetii* infection in participants was based solely on serologic findings as

Address for correspondence: TJ Marrie, Department of Medicine, University of Alberta, 2F1.30 Walter C. Mackenzie Health Sciences Centre, 8440 – 112 St. Edmonton, Alberta T6G 2R7, Canada; fax: 780-407-3132; email: tom.marrie@ualberta.ca

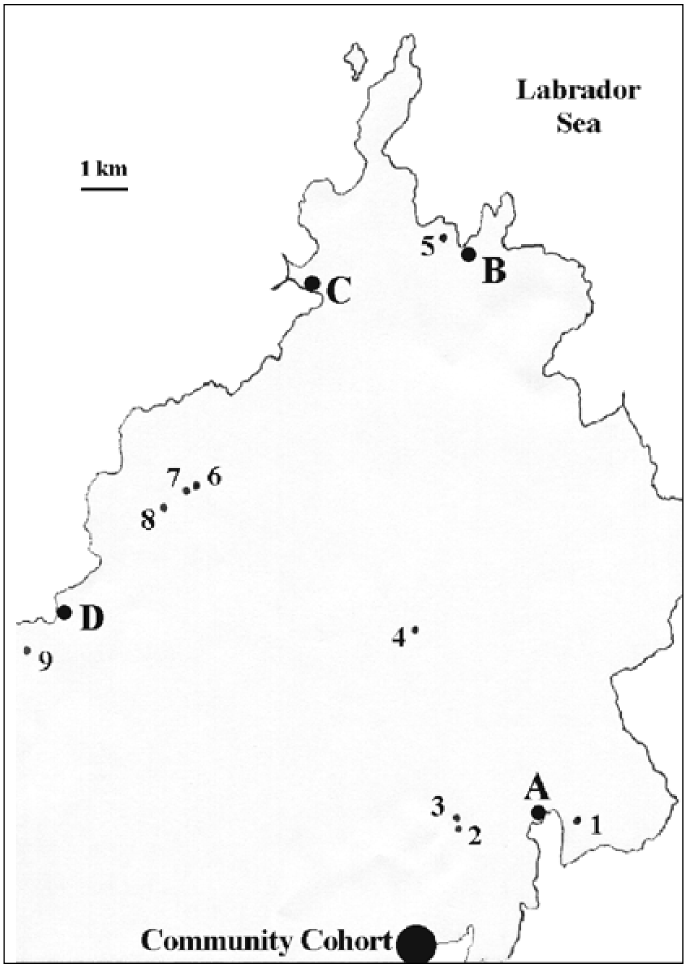

Figure 1. Map of the Newfoundland goat cooperative showing the farms (2-9) in relationship to the surrounding communities (A-D, the area from which the community cohort was derived). (Note: farm 1 is not directly involved with the cooperative.)

described below. In July 1999, follow-up serum samples were obtained to determine further evidence of seroconversion. In addition, 2 weeks earlier, serum was collected from 154 volunteers from adjacent communities (community cohort) and a questionnaire was completed for comparison with the outbreak cohort.

Serum samples were collected in May 1999 from 387 random blood donors, primarily from urban areas. These samples were used to determine the background seroprevalence of *C. burnetii* infection in Newfoundland.

### Source of Animals and Identification of *C. burnetii* Infection in Animals

Although a few locally raised goats were present in the community before the cooperative was established, the eight farms received shipments of goats from Ontario, Nova Scotia, Prince Edward Island, and Maine in the summer and fall of 1998. At the time of the outbreak, 174 goats were within the cooperative, with 10 to 38 animals per herd. Serum samples were obtained from 147 goats to determine the extent of *C. burnetii* infections in the animals.

Serum samples were collected from livestock from other farms throughout Newfoundland to determine the background seroprevalence of Q fever in farm animals in Newfoundland.

### Laboratory Studies

Antibody titers (immunoglobulin G [IgG]) to *C. burnetii* phase I and phase II antigens were determined (15). Antibodies were detected by using indirect immunofluorescence with whole cells of the Nine-Mile strain of *C. burnetii*. An IgG antibody titer of $\geq$1:8 was considered seropositive, indicating prior exposure to *C. burnetii*. Acute *C. burnetii* infection was characterized by a phase II IgG titer of 1:64 or a fourfold rise in titer between two separate serum samples.

Placenta samples from goats were sent to Dr. D. Raoult in France, where they were processed for polymerase chain reaction (PCR) using established protocols (16).

### Epidemiologic Studies

A standardized questionnaire was administered to participants who submitted a serum sample. Demographic data, a detailed history of exposure to goats, clinical history, and symptoms were collected by direct interview. Where available, charts of patients were reviewed to collect additional clinical and laboratory data.

To construct epidemic curves, date of onset of symptoms was considered the date of infection. When this date was unavailable (in asymptomatic cases and participants lacking clinical data), date of infection was based on date of the first serum sample (if it had a diagnostic titer) or the halfway point in those who demonstrated a fourfold rise in antibody titer between acute- and convalescent-phase serum samples.

### Statistical Analysis

Differences between infected and uninfected participants were tested for statistical significance by using the chi-square test for proportions and Student *t* test for means. Independent risk factors for infection were determined by using a backward logistical regression analysis. Variables with a p value of <0.05 on univariate analysis were entered into the regression analysis. All data were analyzed by using SPSS for Windows version 8.0 (SPSS Inc. 1989-1999); results were considered significant when p was <0.05.

### Results

### Clinical Illness in Goats and Humans

Kidding began January 6 and ended April 24, 1999. Although occasionally it was restricted to dedicated pens, most birthing took place in a communal pen on each farm. *Coxiella* was identified in placental samples examined by using electron microscopy and light microscopy (Gimenez stain), and *C. burnetii* DNA was demonstrated in all three placental samples with PCR. A total of 30 abortions were recorded at six of the eight farms. (Some farms had incomplete records.) The first abortion occurred in December before the kidding season began; the others took place between January 14 and April 24, with abortion rates of 16%-22% per farm. There was no relationship between seropositivity in goats and frequency of abortion.

The epidemic curves differed from farm to farm. Evidence of a continuous common source of infection was seen at one farm (Figure 2), while other evidence suggested a point source (Figure 3). The overall epidemic curve suggested a continuous

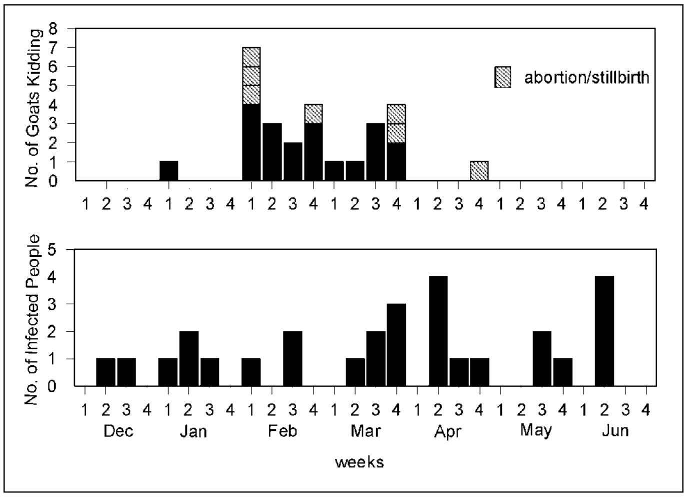

Figure 2. Epidemic curve for farm no. 4, showing the timing of human infection with kidding and abortions.

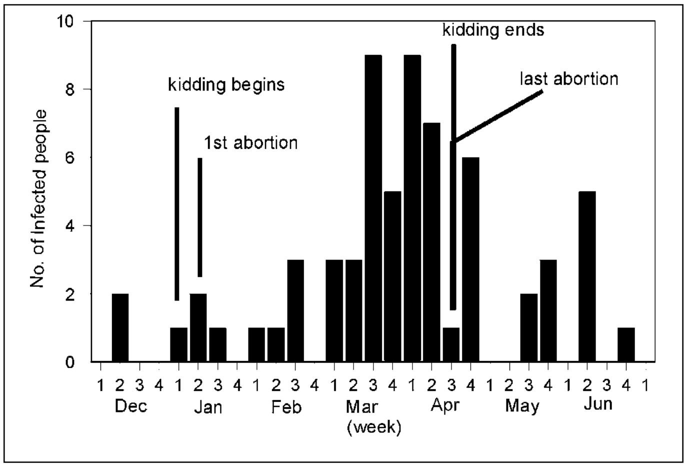

Figure 4. Overall epidemic curve for Q fever outbreak associated with Newfoundland goat cooperative.

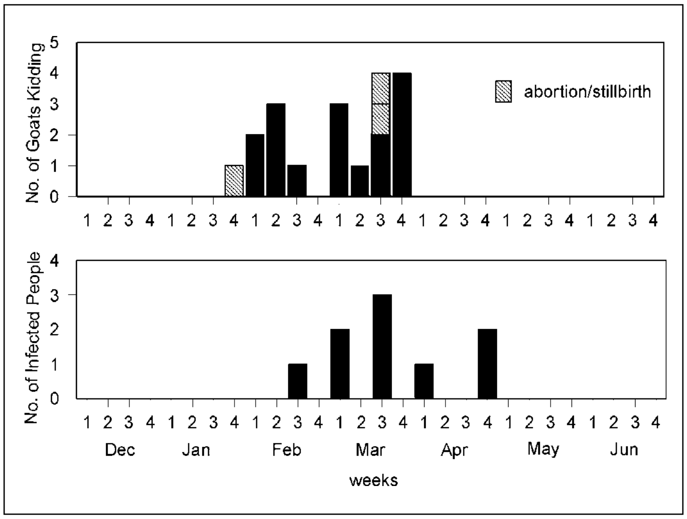

Figure 3. Epidemic curve from farm no. 6, showing the timing of human infection with kidding and abortions.

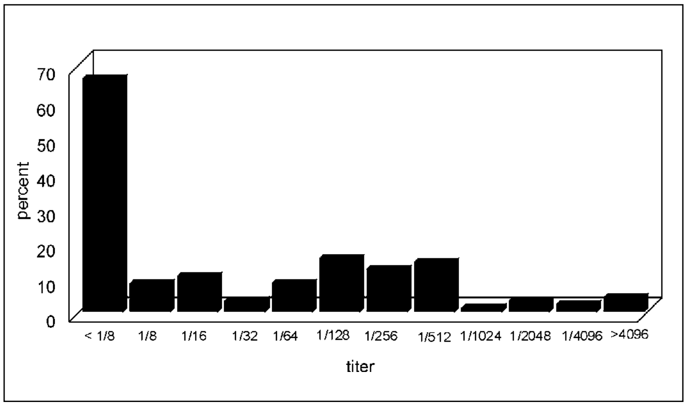

Figure 5. Antibody titers to *Coxiella burnetii* phase II antigen in the outbreak cohort. Of the infected cohort, 24/66 had a fourfold rise in antibodies to phase II (24/66 of the infected cohort had a fourfold rise in antibodies to phase II antigen)

source or reservoir for infection that had a peak during the kidding season (Figure 4).

Illness in goat farmers or their workers was noted in March 1999. Serologic data were available for 179 farmers, workers, and contacts (outbreak cohort). Eighty (44.7%) outbreak cohort participants had antibodies against the phase II antigen. Sixty-six (36.9%) had phase II titers of ≥1:64 or had a fourfold rise in titer, suggesting recent infection (Figure 5). The seroprevalence of infected workers (including farmers) on each farm ranged from 0 (farm 5) to 87.5% (farm 4). In comparison, 35 (22.7%) of 154 community cohort participants were seropositive ($p<0.001$), and 2 (1.3%) had titers of antibodies to phase II antigen of >1:64 (Figure 6). Seroprevalence in blood donors (8.3%) (Table 1) was significantly lower than that of the control ($p<0.001$) and outbreak ($p<0.001$) cohorts. Five blood donors (1.3%) had titers to phase II antigen ≥1:64.

Questionnaires were completed by 146 (81.6%) farm workers or contacts who provided blood samples. The remaining 33 could not be reached for questioning. Of the 146 participants, 9 (6.2%) were farmers, 58 (39.7%) were workers, and 79 (54.1%) were contacts. Demographic data were collected (Table 2). The infected and noninfected groups had equal numbers of men and women. Infected persons tended to be slightly older, were more likely to have been ill in the past 2 months (odds ratio [OR] 3.53), and to have visited their doctor during that time (OR 3.13). Symptoms associated with infection included sweats, chills, headache, weight loss, malaise, fever, fatigue, myalgias, dyspnea, nausea, and diarrhea (Table 2).

The incubation period for Q fever was difficult to determine as most people had many contacts with goats. However, three persons could recall the date of a specific high-risk activity such as assisting with the delivery of a stillborn kid. Incubation periods for these three persons were 21, 31, and 36 days.

A family physician performed clinical laboratory tests on 25 of the infected persons. Four (16%) of these had transaminase levels >1.5x, the upper limit of normal. Eight had X rays; one had pneumonia.

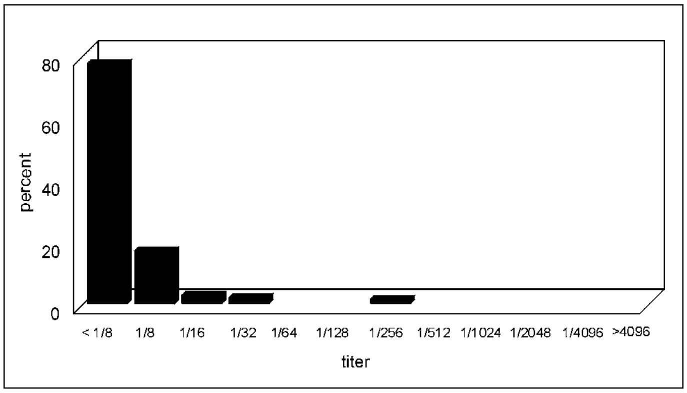

Figure 6. Antibody titers to *Coxiella burnetii* phase II antigen in the community cohort.

Table 1. Seroprevalance of antibodies to *Coxiella burnetii* phase II antigen in random blood donors from Newfoundland

| Region | Seropositivity no. (%) |
|---|---|
| St. John's Center | 12/155 (7.7) |
| Cornerbrook | 1/31 (3.2) |
| St. John's | 7/57 (12.3) |
| Norman's Cove | 4/40 (10.0) |
| Foxtrap | 6/71 (8.5) |
| Conception Bay Central | 2/33 (6.1) |
| Total | 32/387 (8.3) |

Table 2. Demographic features and symptoms associated with human *Coxiella burnetii* infection in Newfoundland outbreak

| Features and symptoms | Infected no. (%) | Noninfected no. (%) | Odds ratio (95% CI) |
|---|---|---|---|
| Male | 31/58 (53.4) | 46/88 (52.3) | 1.05 (0.154-2.04) |
| Female | 27/58 (46.6) | 42/88 (47.7) | |
| Mean age | 38.48±11.76 | 33.47±19.34 | p=0.054 |
| Sick in the past 2 months | 49/60 (81.7) | 48/86 (55.8) | 3.53 (1.62–7.70) |
| Symptoms | | | |
| Sweats | 11/12 (91.7) | 3/9 (33.3) | 22.0 (1.86–260.5) |
| Chills | 12/14 (85.7) | 4/10 (40.0) | 9.00 (1.27–63.90) |
| Headache | 43/60 (71.7) | 20/78 (25.6) | 7.34 (3.44–15.64) |
| Malaise | 44/59 (74.6) | 28/77 (36.4) | 5.13 (2.43–10.84) |
| Weight loss | 8/31 (25.8) | 3/43 (7.0) | 4.64 (1.12–19.24) |
| Fever | 40/60 (66.7) | 25/79 (31.6) | 4.32 (2.11–8.84) |
| Fatigue | 39/58 (67.2) | 27/79 (34.2) | 3.95 (1.93–8.11) |
| Myalgias | 26/58 (44.8) | 15/76 (19.7) | 3.30 (1.54–7.11) |
| Dyspnea | 19/60 (31.7) | 12/78 (15.4) | 2.55 (1.12–5.79) |
| Nausea | 30/60 (50.0) | 22/80 (27.5) | 2.24 (1.30–5.34) |
| Diarrhea | 23/60 (38.3) | 18/81 (22.2) | 2.18 (1.04–4.55) |
| Cognitive[a] | 14/30 (46.7) | 4/15 (26.7) | 2.41 (0.62–9.29) |
| Sputum | 13/46 (22.0) | 11/78 (14.1) | 1.72 (0.71–4.18) |
| EOM pain | 8/15 (53.3) | 4/9 (44.4) | 1.43 (0.27–7.52) |
| Neck stiffness | 16/22 (72.7) | 9/14 (64.3) | 1.43 (0.27–7.52) |
| Vomiting | 12/60 (20.0) | 15/81 (18.5) | 1.10 (0.47–2.56) |
| Pleuritic pain | 8/57 (14.0) | 10/76 (13.2) | 1.08 (0.40–2.93) |
| Cough | 22/60 (36.7) | 28/81 (34.6) | 1.10 (0.55–2.20) |
| Loss of libido | 13/32 (40.6) | 5/12 (41.7) | 0.96 (0.25–3.38) |

[a]Cognitive problems, including changes in concentration, memory, or temper. EOM, extra-ocular eye movement; CI, confidence intervals.

### Risk Factors for Q Fever

Risk factors associated with human infection on univariate analysis included being a farmer, milking goats, assisting with kidding, handling placentas, shoveling manure, having direct contact with goats, eating cheese made from goat milk, petting goats, feeding goats, being a worker, smoking tobacco, and drinking alcohol (Table 3). When only a multivariate analysis was used, the following were significant risk factors for infection with *C. burnetii*: contact with the placenta (p<0.001), smoking history (p=0.001), and eating cheese made from goat milk (p= 0.022). Both infected persons in the community cohort also had direct contact with goats.

Overall, 82 (55.8%) of the 147 goats were seropositive (range from 10% to 100%, depending on the farm); antibody titers ranged from 1:8 to >1:4,096. Although 8 (50%) of 16 goats from other areas in eastern Newfoundland had antibodies to *C. burnetii*, the highest titer was 1:16. In contrast, titers in goats in the outbreak ranged from 1:8 to >1:4,096. In the goats in the cooperative, 63 (43%) and 30 (20%), respectively, had an antibody titer of ≥1:64 to phase I and phase II antigen. Correlation between *C. burnetii* infection in goats and the geographic origin of the animals or determination of a relationship between seropositivity of goats and the number of persons infected on each farm was not feasible because of insufficient data.

Table 3. Exposure risks associated with *Coxiella burnetii* infection in the Newfoundland outbreak

| Risk factors | Infected no.(%) | Noninfected no.(%) | Odds ratio (95% CI) |
|---|---|---|---|
| Visited a barn | 57/60 (95.0) | 63/86 (73.3) | 6.94 (1.98–24.34) |
| Direct contact with goats | 54/60 (90.0) | 54/86 (62.8) | 5.33 (2.06–13.79) |
| Milking | 19/60 (31.7) | 3/85 (3.5) | 12.67 (3.54–45.29) |
| Assisting with kidding | 29/60 (48.3) | 6/85 (7.1) | 12.32 (4.66–32.57) |
| Handling placenta[a] | 31/60 (51.6) | 7/86 (8.1) | 12.06 (4.79–30.39) |
| Shoveling manure | 37/60 (61.7) | 19/84 (22.6) | 5.50 (2.65–11.41) |
| Feeding | 39/57 (68.4) | 29/85 (34.2) | 4.17 (2.06–8.43) |
| Petting goats[a] | 52/60 (86.7) | 51/85 (60.0) | 4.33 (1.83–10.26) |
| Farmer | 8/60 (13.3) | 1/85 (1.2) | 12.92 (1.57–106.32) |
| Farm worker | 34/60 (56.7) | 24/86 (27.9) | 3.38 (1.67-6.77) |
| Household contact, visited farm | 13/60 (21.7) | 35/85 (41.2) | 0.40 (0.19-0.84) |
| Household contact, no farm visit | 2/60 (3.3) | 23/85 (27.1) | 0.09 (0.02–0.41) |
| Ate goat cheese[a] | 17/60 (28.3) | 6/86 (7.0) | 5.27 (1.94–14.35) |
| Smoked[a] | 36/58 (62.1) | 28/84 (33.3) | 3.27 (1.63–6.58) |
| Drank alcohol | 37/57 (64.9) | 38/84 (45.2) | 2.08 (1.04–4.13) |
| Have liver problems | 5/53 (9.4) | 2/72 (2.8) | 3.65 (0.68-19.57) |
| Have cats | 25/59 (42.4) | 30/86 (34.9) | 1.37 (0.70–2.71) |
| Drink goat milk | 19/60 (31.7) | 27/86 (31.4) | 1.01 (0.50–2.06) |

[a]By logistic regression model, the following were statistically significant: Contact with placenta (p <0.001); smoking history (p=0.001); eating goat cheese (p=0.022); and petting goats (p=0.055).

### Conclusion

Goats have been implicated in outbreaks of Q fever in the United States, Ontario, Bulgaria, Slovakia, Greece, and Australia, and have replaced sheep and cattle as the most common source of human infection with *C. burnetii* in Bulgaria (17-19). An estimated 20% of Ontario's dairy goat population have antibodies to *C. burnetii* (20).

The incubation period and clinical illness seen in the Newfoundland outbreak were consistent with those reported for other outbreaks (5,15,21,22). The most common manifestation of *C. burnetii* infection was an acute febrile illness. Although dyspnea was an associated feature of our outbreak, only one of eight patients with X rays had pneumonia. This is in contrast to what is typically seen in Nova Scotia, where *C. burnetii* pneumonia is common after exposure to infected parturient cats (15,23).

Although the patients reported here are the first documented cases of Q fever in Newfoundland, serologic results from blood donors suggest that infection with this organism is present elsewhere in this province but goes unrecognized. The seroprevalence of *C. burnetii* in Newfoundland blood donors (8.3%) is consistent with results from blood donors in other Atlantic Canadian provinces (24,25). The higher seroprevalence in the population from communities surrounding the outbreak area (22.7%) could be due to their close proximity to the outbreak area or may reflect a difference in prevalence, which is often higher in rural areas (11).

The eight farms in the cooperative house their goats in small, uninsulated, naturally ventilated barns, many of which have concrete floors. The winter and spring months in Newfoundland can be quite cold, so to provide better insulation the hay spread on floors of the pens is packed down instead of being disposed of regularly. The resulting "manure pack" would be heavily contaminated by *C. burnetii* in feces, urine, and products of conception. Removing the bedding would generate aerosols containing *C. burnetii*. *Coxiella* is very hardy and resists desiccation, remaining viable in soil for several years (26).

Contaminated hay and manure were also spread on the rocky ground to fertilize small pastures next to the barns. This method of disposal represents potential sources of exposure for surrounding communities. Inhalation of *C. burnetii* from contaminated environments is well documented, and contaminated fields and roads often serve as reservoirs for airborne spread of *C. burnetii* (5,18,22,27,28). Studies from Europe demonstrate that wind can spread *C. burnetii* >18 km from its source (29). These newly developed pastures in the Newfoundland cooperative may explain the higher seroprevalence rate in the community cohort compared with that in blood donors from across the province.

Kidding took place in isolated pens but also occurred in communal areas of the barn. Placental tissue and aborted kids were disposed of by incineration or burial. Although the workers usually did not handle the placenta, they would often help clean and dry newborn goats covered in amniotic fluid without the protection of masks or gloves. Exposure to the birth products of infected animals has been consistently shown to be a risk factor in other Q fever outbreaks (28). Given that *Coxiella* is shed in high numbers in birth products (7) and aerosolization of the microorganism can persist for days after parturition, despite immediate removal of the highly infectious placenta (30), it is not surprising that exposure to the placenta was an independent risk factor for infection ($p<0.001$).

In our study, smoking tobacco was an independent risk factor for infection ($p=0.001$). This could be due to contaminated hands touching cigarettes, resulting in ingestion of *Coxiella*. Smoking does impair pulmonary host defenses and thus may have contributed to this finding (31). In addition, some barns did not have running water and washrooms until late in the spring, contributing to poor hygienic practices in some instances.

The role of drinking unpasteurized milk in *C. burnetii* infection is controversial. *C. burnetii* has been recovered from milk from infected cows and goats and from butter (17,32). Epidemiologic studies suggest that ingestion of unpasteurized milk has been a source of *Coxiella* infection for humans (6,17,33). Experimental evidence to support a causal relationship is sparse. Asymptomatic seroconversion and infection were noted in inmates fed raw milk from a Q fever infected herd (33). In another study, volunteers who drank naturally infected unpasteurized milk did not develop symptoms or an immunologic response to suggest infection (34). These authors suggest that the lack of seroconversion in their study may have been related to exposure to a different *Coxiella* strain than the one that caused infection in the inmate population (33,34). Pasteurization will effectively kill *Coxiella* in raw milk (35). However, in our study, ingestion of cheese made from pasteurized goat milk was identified as an independent risk factor for infection ($p=0.022$) even though consumption of goat milk itself was not associated with an increased risk of infection (OR 1.07). This is the first time a pasteurized dairy product has been implicated in an outbreak of Q fever. However, 21 (14%) of 154 members of the community cohort ate the product but were not infected. The reason for the association between ingesting goat cheese and developing Q fever is not clear and suggests further study is needed. At present, this is an epidemiologic association only, as *C. burnetii* has not been recovered from the goat cheese.

In Canada, *C. burnetii* infection is not a reportable disease in animals (36). Serbezov (19) suggests that "goats may pose a threat to human health as a source of *C. burnetii* infection in every country in which they are raised extensively and are in close contact with humans." Goats in the Newfoundland cooperative originated from four different sources—Maine, Nova Scotia, Prince Edward Island, and Ontario. Although the sale and movement of infected animals have been implicated in spreading the disease (4), there was no relationship between the seroprevalence rate of goats originating from one area compared to another, making it difficult to determine if one group of imported animals was responsible for initiating the outbreak. However, goats tend to remain chronically infected, and once infection is established it can spread rapidly through the remaining herds (37). Once *C. burnetii* infection was identified in the herd, only four goats on one farm in the cooperative were treated with antibiotics.

These are the first cases of Q fever in Newfoundland. The small barns and poor ventilation created confined conditions and an environment that facilitated infection. Although exposure to goats and eating unpasteurized milk have been implicated in causing *C. burnetii* infection in the past, this is the first time that a product made from pasteurized milk has been associated epidemiologically as a risk factor. Outbreaks of Q fever in research institutions as a result of exposure to infected parturient sheep and goats has led to number of recommendations (38-41). These recommendations include using only *C. burnetii*-seronegative animals in research; vaccinating seronegative animals; using protective clothing and masks while working with animals (especially pregnant ones); restricting access to animals; properly decontaminating surfaces with formalin or bleach solutions; properly disposing of waste by incineration; and using caution, culling, confinement, or chemotherapy in herds with a rate of >20% seropositivity containing animals with titers ≥1:32.

Some of these measures are difficult to carry out on a dairy farm; however, since data suggest that human infection can be prevented by vaccination with formalin-inactivated phase I *C. burnetti*, persons at risk from occupational exposure should be offered the vaccine (41).

Our experience raises many questions about management of *C. burnetii* outbreaks in the dairy industry, the interprovincial sale and movement of domestic ungulates, and the need for discussion between public health practitioners and the dairy industry on control of this highly infectious organism.

### Acknowledgments

We thank E. Dumka, J. Norman, F. Priddle, M. Hayes, S. Burbridge, D. Waag, and H. Whitney for their cooperation and assistance.

Dr. Hatchette is a fellow in infectious diseases at Dalhousie University.

### References

1. Kaplan MM, Bertagna P. The geographical distribution of Q fever. Bull World Health Org 1955;13:829-60.
2. Marrie TJ, Embil J, Yates L. Seroepidemiology of *Coxiella burnetii* among wildlife in Nova Scotia. Am J Trop Med Hyg 1993;49:613-15.
3. Marrie TJ, Van Buren J, Fraser J, Haldane EV, Faulkner RS, Williams JC, et al. Seroepidemiology of Q fever among domestic animals in Nova Scotia. Am J Public Health 1985;75:763-6.
4. Luoto L, Pickens EG. A resume of recent research seeking to define the Q-fever problem. Am J Hyg 1961;74:43-9.
5. Dupuis G, Petite J, Peter O, Vouilloz M. An important outbreak of human Q fever in a Swiss alpine valley. Int J Epidemiol 1987;16:282-7.
6. Fishbein DB, Raoult D. A cluster of *Coxiella burnetii* infections associated with exposure to vaccinated goats and their unpasteurized dairy products. Am J Trop Med Hyg 1992;47:35-40.
7. Abinanti FR, Lennette EH, Winn JF, Welsh HH. Q fever studies XVVII. Presence of *Coxiella burnetii* in the birth fluids of naturally infected sheep. Am J Hyg 1953;58:358-88.
8. Tigertt WD, Benenson AS, Gochenour WS. Airborne Q fever. Bacteriol Rev 1961;25:285-93.
9. Ormsbee R, Peacock M, Gerloff R, Tallent G, Wike D. Limits of rickettsial infectivity. Infect Immun 1978;19:239-45.
10. Moore JD, Barr BC, Daft BM, O'Connor MT. Pathology and diagnosis of *Coxiella burnetii* infection in a goat herd. Vet Pathol 1991;28:81-4.
11. Tissot Dupont H, Raoult D, Brouqui P, Janbon F, Peyramond D, Weiller PF, et al. Epidemiologic features and clinical presentation of acute Q fever in hospitalised patients: 323 French cases. Am J Med 1992;93:427-34.
12. Raoult D, Tissont-Dupont H, Foucault C, Gouvernet J, Fournier PE, Bernit E, et al. Q fever 1985-1998. Clinical and epidemiologic features of 1383 infections. Medicine 2000;79:109-23.
13. Fournier PE, Marrie TJ, Raoult D. Diagnosis of Q fever. J Clin Microbiol 1998;36:1823-34.
14. Peacock MG, Philip RN, Williams JC, Faulkner RS. Serologic evaluation of Q fever in humans: Enhanced phase I titres of immunoglobulins G and A are diagnostic for Q fever endocarditis. Infect Immun 1983;41:1089-98.
15. Marrie TJ, MacDonald A, Durant H, Yates L, McCormick L. An outbreak of Q fever probably due to contact with a parturient cat. Chest 1988;93:98-103.
16. Willems HD, Thiele D, Frolich-Ritter R, Krauss H, et al. Detection of *Coxiella* in cow's milk using the polymerase chain reaction. J Vet Med 1994;41:580-7.
17. Lennette EH, Clark WH. Observations on the epidemiology of Q fever in northern California. JAMA 1951;145:306-9.
18. Spicer AJ, Crowthier RW, Vella EE, Bengtsson E, Miles R, Pitzolis G. Q fever and animal abortion in Cyprus. Trans R Soc Trop Med Hyg 1977;71:16-20.
19. Serbezov V, Kazar J, Novkirishki V, Gatcheva N, Kovacova E, Voynova V. Q fever in Bulgaria and Slovakia. Emerg Infect Dis 1999;5:388-94.
20. Lang GH. Serosurvey of *Coxiella burnetii* infection in dairy goat herds in Ontario. A comparison of two methods of enzyme-linked immunosorbent assay. Can J Vet Res 1988;52: 37-41.
21. Clark WH, Lennette EH, Railsback OC, Romer MS. Q-fever in California vii. Clinical features in one hundred eighty cases. Arch Intern Med 1951;88:155-67.
22. Derrick EH. The course of infection with *Coxiella burnetii*. Med J Aust 1973;1:1051-7.
23. Marrie TJ, Durant H, Williams JC, Mintz E, Waag DM, et al. Exposure to parturient cats: A risk factor for acquisition of Q fever in Maritime Canada. J Infect Dis 1988;158:101-8.
24. Marrie TJ, VanBuren J, Faulkner RS, Haldane EV, Williams JC, Kwan C. Seroepidemiology of Q fever in Nova Scotia and Prince Edward Island. Can J Microbiol 1984;30:129-34.
25. Marrie TJ. Seroepidemiology of Q fever in New Brunswick and Manitoba. Can J Microbiol 1988;34:1043-5.
26. Reimer LG. Q fever. Clin Microbiol Rev 1993;6:193-8.
27. Tissot-Dupont H, Torres S, Nezri M, Raoult D. Hyperendemic focus of Q fever related to sheep and wind. Am J Epidemiol 1999;150:67-74.
28. Hart RJC. The epidemiology of Q fever. Postgrad Med J 1973;49:535-8.
29. Hawker JI, Ayres JG, Blair I, Evans MR, Smith DL, Smith EG, et al. A large outbreak of Q fever in the West Midlands: windborne spread into a metropolitan area? Commun Dis Public Health 1998;1:180-7.
30. Welsh HH, Lennette EH, Abinanti FR, Winn JF. Airborne transmission of Q fever: The role of parturition in the generation of infective aerosols. Ann NY Acad Sci 1958;70:528-40.
31. Daniele RP, Dauber JH, Altose MD, Rowlands DT, Gorenberg DJ. Lymphocyte studies in asymptomatic cigarette smokers: A comparison between lung and peripheral blood. Am Rev Respir Dis 1977;116:997-1005.
32. Biberstein EL, Behymer DE, Bushnell R, Crenshaw G, Riemann HP, Franti CE. A survey of Q fever (*Coxiella burnetii*) in California dairy cows. Am J Vet Res 1974;35:1577-82.
33. Benson WW, Brock D, Mather J. Serologic analysis of a penitentiary group using raw milk from a Q fever infected herd. Public Health Rep 1963;78:707-10.
34. Krumbiegel ER, Wisniewski HJ. Consumption of infected raw milk by human volunteers. Arch Environ Health 1970;21:63-5.

35. Enright HB, Sadler WW, Thomas RC. Pasteurisation of milk containing the organism of Q fever. Am J Pub Health 1957;47:695-700.
36. Lang GH. Serosurvey of the occurrence of *Coxiella burnetii* in Ontario cattle. Can J Public Health 1988;79:56-9.
37. Wisniewski HJ, Krumbiegel ER. Q fever in the Milwaukee area—I. Q fever in Milwaukee. Arch Environ Health 1970;21:58-65.
38. Simor AE, Brunton JL, Salit IE, Vellend H, Ford-Jones L, Spence LP. Q fever: hazard from sheep used in research. Can Med Assoc J 1984;130:1013-6.
39. Graham CJ, Yamauchi T, Rountree P. Q fever in animal laboratory workers: An outbreak and its investigation. Am J Infect Control 1989;17:345-8.
40. Perry S, Dennie CJ, Coblentz CL, Cleland S. Minimizing the risk of Q fever in the hospital setting. Can J Infect Control 1994;9:5-8.
41. Maurin R, Raoult D. Q fever. Clin Microbiol Rev 1999;12:518-53.
